# Strengthening the Swiss health system by improving organizational health literacy: a practice-approach

**DOI:** 10.1093/eurpub/ckac129.381

**Published:** 2022-10-25

**Authors:** S De Gani, AS Bilgeri

**Affiliations:** Careum Center for Health Literacy, Careum Foundation, Zuerich, Switzerland

## Abstract

**Issue:**

Healthcare organizations represent vital points of contact and disseminators for people to find, understand, assess, and apply health information, and may thereby strengthen people's health literacy (HL). From a system perspective, healthcare organizations provide essential practices and structures to foster population health beyond their medical-based services.

**Description of the problem:**

Despite increasing research on the promising role of organizational health literacy (OHL) the current Swiss healthcare system provides insufficient support for healthcare organizations to address HL. In response, the Careum Foundation in Zurich recently launched a Center for Health Literacy to promote OHL - among other HL initiatives. A first step was a practice-oriented collaboration project with the Department of Health of the canton of Zurich, which started in 2019 to assess, implement, and improve HL in primary care organizations. Therefore, a self-assessment tool for OHL is being developed, implemented, and evaluated. In addition, the center started to shed light on HL in Switzerland, on HL and necessary competencies of health professionals. Moreover, it is investing in communication and expertise on OHL, and developing first ideas regarding health literate hospitals.

**Results:**

A first systematic evaluation of the self-assessment tool has demonstrated a significant potential to improve OHL, organizational development, and teambuilding processes in healthcare organizations. Connected through (inter)national networks, the Center for Health Literacy launches a practice approach to understand OHL as both medium and outcome of a health literate population.

**Lessons:**

We have learned that practice-oriented OHL initiatives can provide promising approaches to strengthen both population health and organizational development processes.

